# Long non-coding RNA RP11-284P20.2 promotes cell proliferation and invasion in hepatocellular carcinoma by recruiting EIF3b to induce c-met protein synthesis

**DOI:** 10.1042/BSR20200297

**Published:** 2020-03-12

**Authors:** Qin-Liang Fang, Jian-Yin Zhou, Yu Xiong, Cheng-Rong Xie, Fu-Qiang Wang, Yun-Tong Li, Zhen-Yu Yin, Guo-Hui Luo

**Affiliations:** 1 Department of Hepatobiliary Surgery, Zhongshan Hospital of Xiamen University, Hubin South Road, No. 201-209, Siming District, Xiamen 361004, China; 2 Fujian Provincial Key Laboratory of Chronic Liver Disease and Hepatocellular Carcinoma, Zhongshan Hospital of Xiamen University, Hubin South Road, No. 201-209, Siming District, Xiamen 361004, China; 3 Department of Nephrology, Zhongshan Hospital of Xiamen University, Hubin South Road, No. 201-209, Siming District, Xiamen 361004, China

**Keywords:** c-met protein, EIF3b, hepatocellular carcinoma, RP11-284P20.2, translation

## Abstract

A newly identified lncRNA designated as RP11-284P20.2 has been identified to be up-regulated in hepatocellular carcinoma (HCC), but its role in HCC remain poorly understood. Quantitative PCR and immunocytochemical analysis were performed using the HCC tissues to identify the potential interaction partners of RP11-284P20.2. Moreover, RP11-284P20.2 was knocked down in HCC cell lines, HepG2 and SMMC7721, to investigate the influence of this lncRNA on cell growth properties. Additionally, RNA fluorescence *in situ* hybridization and immunofluorescence, RNA immunoprecipitation, and RNA pull-down assays were performed to determine the interaction of RP11-284P20.2 with *c-met* mRNA and eukaryotic translation initiation factor 3b (EIF3b). Silencing RP11-284P20.2 inhibited cell viability, migration, invasion, and colony formation, and increased apoptosis. Overexpression of c-met abolished these effects of RP11-284P20.2 in HCC cells. Histopathological examination showed that HCC tissues with high RP11-284P20.2 expression had higher c-met protein level than that in HCC tissues with low RP11-284P20.2 expression. However, there was no positive correlation between the expression levels of RP11-284P20.2 and *c-met* mRNA. RP11-284P20.2 knockdown led to a decease in c-met protein expression level, but did not affect the *c-met* mRNA expression level. These data suggest that RP11-284P20.2 regulates c-met protein expression level, which is independent of *c-Met* mRNA expression level. It was also confirmed that RP11-284P20.2 has high affinity toward both *c-met* mRNA and EIF3b protein, and hence RP11-284P20.2 probably recruits EIF3b protein to *c-met* mRNA and further facilitates its translation. RP11-284P20.2 promotes cell proliferation and invasion in hepatocellular carcinoma by recruiting EIF3b to induce c-met protein synthesis.

## Introduction

Hepatocellular carcinoma (HCC) is the most commonly diagnosed liver cancer, which accounts for almost 90% of liver cancer-related morbidity worldwide [1]. Moreover, despite the advancement in standardized treatment strategies such as surgical resection, HCC results in >700,000 mortalities every year [1,2]. HCC is a typical inflammation-related cancer, which is an inflammation caused by chronic hepatitis B and C infections. Such inflammation can induce changes in the gene expression pattern in hepatocytes, thereby promoting the transformation of hepatocytes to tumor cells [1,2]. Growing number of studies suggests that the occurrence of HCC is associated with an aberrant activation of c-met, a transmembrane tyrosine kinase receptor of hepatocyte growth factor (HGF). The primary function of the c-met pathway is largely restricted to organ morphogenesis during development, with a limited role in damage repair and regeneration of tissue in adults [3,4]. However, abnormal activation of the HGF/c-met pathway has been found to stimulate cell proliferation, survival, migration, invasion, angiogenesis, and morphogenic differentiation. These pleiotropic effects of c-met are mainly associated with the interaction of c-met cytoplasmic domain with multiple proteins, such as epithelial growth factor receptor, chemokine receptor 4, vascular endothelial growth factor receptor 2, and fibroblast growth factor receptor [3,4].

Long non-coding RNAs (lncRNAs) are classified as the non-coding RNAs (ncRNAs) that are usually >200 nucleotides in length. lncRNAs were initially considered as “dark matter” in the transcription process; however, a large number of subsequent studies revealed that lncRNAs actively participate in multiple biological processes such as transcription, epigenetic regulation, mRNA stabilization as well as translation, and thus can drastically modulate the expression of various genes [5]. However, aberrant expression of some lncRNAs and their procarcinogenic effects have been reported in HCC and other types of cancers. For example, lncRNA–PDPK2P was highly expressed in HCC tissues, and its up-regulation was clinically associated with a larger tumor embolus, low differentiation rate, and poor survival. Mechanistically, it was identified that lncRNA–PDPK2P interacts with PDK1 and promotes HCC progression through the PDK1/AKT/caspase 3 signaling pathway [6].

In our laboratory, we recently performed high-throughput sequencing to identify the lncRNAs, which exhibit significant difference in their expression levels between the HCC and the adjacent paracancerous tissues. We discovered that a newly identified lncRNA named RP11-284P20.2 is up-regulated in HCC tissues, and more importantly is positively correlated with c-met protein expression level. *RP11-284P20.2* is a pseudogene, which is located at chr9:13986175-13987909 and spans up to 1735 nucleotides (nt) in length. Since RP11-284P20.2 has never been reported to be associated with any type of cancer or other diseases, the regulatory effects of this novel lncRNA on the progression of HCC and c-met expression are completely unknown. Thus, in the present study we aimed to explore the functional role and downstream mechanism of RP11-284P20.2 in HCC cells.

## Material and methods

### Collection of clinical samples

HCC tissues and the adjacent paracancerous tissue samples (2 cm) were collected from 27 HCC patients who underwent surgical treatment at the Zhongshan Hospital of Xiamen University from a time period of January to November 2018. All the participating patients provided the written informed consent for using their tissues in this experimental study. The present study was carried out with the approval of the Ethics committee of the Zhongshan Hospital of Xiamen University. All these cases were histologically verified, and their clinical stage was assigned at the time of diagnosis using the TNM staging system of the American Joint Committee on Cancer. All the participating HCC patients had a history of chronic hepatitis B and none of them had received transcatheter arterial chemoembolization, immunization, or targeted anti-cancer therapy before the surgical operation.

### Immunocytochemical analysis

Paraffin-embedded sections of the HCC tissue specimens were deparaffinized and heated in 10 mM citrate buffer (pH 6.0) at 97°C for 20 min to retrieve the antigens. Tissue sections were incubated with anti-c-met antibody (1:1000 dilution, ab74217; Abcam, Cambridge, MA, U.S.A.) at 4°C for overnight, followed by horseradish peroxidase (HRP)-labeled anti-rabbit IgG (1:200; Abcam) at room temperature for 30 min. Each section was immersed in 500 µl of working solution of diaminobenzidine for 3–10 min.

### Cell lines and culture conditions

Six different HCC cells lines, including HepG2, Huh7, SMMC7721, SUN-398, SUN-449, and Bel-7402, were purchased from the American Type Culture Collection (Manassas, VA, U.S.A.). All the cell lines were maintained in Dulbecco’s modified Eagles’ medium (DMEM; Invitrogen, Carlsbad, CA, U.S.A.) supplemented with 10% fetal bovine serum (HyClone, Logan, Utah, U.S.A.) and 1% penicillin/streptomycin at 37°C in a humidified incubator supplied with a mixture of 95% air and 5% CO_2_.

### Quantitative real-time PCR analysis (qRT-PCR)

To quantify the expression levels of RP11-284P20.2 and *c-met* mRNA, total RNA from the tissues and cells was extracted using Trizol reagent (Invitrogen; Thermo Fisher Scientific, Inc.). RNA was reverse transcribed to generate cDNA using the Quantitect Reverse Transcriptase kit (Qiagen GmbH, Hilden, Germany). We then performed qPCR in a 7300 Sequence Detection System (Applied Biosystems, Foster City, CA, U.S.A.) using the SYBR® Green PCR kit (Applied Biosystems). Each reaction had 6 μl of cDNA, 0.22 μl of forward primer and reverse primer at 10 μM final concentration, 7.5 μl of PCR Master Mix, and 1.06 μl of deionized (Milli-Q grade) water. The sequence of the primers used is as follow: *c-met* forward primer: 5′-AGCAATGGGGAGTGTAAAGAGG-3′, *c-met* reverse primer: 5′-CCCAGTCTTGTACTCAGCAAC-3′. *RP11-284P20.2* forward primer: 5′-AGACCATTCACCATGGAGTTCA-3′, *RP11-284P20.2* reverse primer: 5′-AACTGATGACGAAGGCCAGG-3′. *GAPDH* forward primer: 5′-GGGTGTGAACCATGAGAAGT-3′, *GAPDH* reverse primer: 5′-TGAGTCCTTCCACGATACCAA-3′. The expression values are represented as the difference in *C*t values normalized to GAPDH for each sample and the relative RNA expression was calculated using the 2^−ΔΔT^ method.

### Western blot analysis

HepG2 and SMMC7721 cells were solubilized in RIPA lysis buffer (Invitrogen; Thermo Fisher Scientific, Inc.) to extract total protein. After boiling for 5–10 min, the protein samples extracted from different cell suspensions were separated by SDS-PAGE and then transferred onto a nitrocellulose membrane. The membrane was blocked with 5% non-fat dry milk prepared in Tris-buffer and incubated with the primary antibodies directed against c-met (1:1000 dilution; Santa Cruz Biotechnology, Inc., Santa Cruz, CA, U.S.A.) for 1 h at room temperature. The membrane was then incubated with horseradish peroxidase-conjugated anti-mouse IgG secondary antibody (Santa Cruz Biotechnology, Inc.), for 4 h at room temperature. The blot was developed using The ECL™ Western Blotting Detection Reagent (GE Healthcare) and the proteins were visualized by enhanced chemiluminescence (Amersham Biosciences).

### Transient transfection assay

HepG2 and SMMC7721 cells were seeded in a 6-well plate and cultured until the cells achieved 70% confluence. Short-interfering RNAs targeting the lncRNA RP11-284P20.2 and the non-targeting sequence (Scrambled RNA) were designed and synthesized by GenePharma (Shanghai, China). To increase c-met protein level in HepG2 and SMMC7721 cells, the sequence of the CDS region of *c-met* gene (NM_001127500, https://www.ncbi.nlm.nih.gov/nuccore/NM_001127500.3) was also synthesized by GenePharma Company and inserted into an expression vector (pEGFP-C1). The empty vector without the *c-met* gene sequence was used as the negative control. The cells were transfected with the above-mentioned siRNAs and *c-met* expression vector or empty plasmid using Lipofectamine 2000 (Thermo Fisher Scientific, Bridgewater, NJ, U.S.A.) according to the manufacturer’s instructions. Alterations in the expression of RP11-284P20.2 and *c-met* mRNA in HepG2 and SMMC7721 cells were confirmed by qPCR analysis before conducting further experiments.

### Cell viability assay

The HCC cells were seeded into the wells of 96-well plates at a concentration of 1 × 10^5^ cells per well. In total, 100 μl of the cell suspension prepared in culture medium was inoculated in each well and the plate was incubated for 24 h. In total, 10 μl CCK-8 reagent (Dojindo Molecular Technologies, Inc., Tokyo, Japan) was added to each well, just 1 h before the end of incubation period. The absorbance value was measured for each well at OD 490 nm using an enzyme immunoassay analyzer.

### Flow cytometric analysis

The HCC cells were dual-stained with Alexa Fluor 488–Annexin V and propidium iodide (PI) using an Annexin V-fluorescein isothiocyanate / PI Apoptosis Detection Kit (Kaiji Biological Inc., Nanjing, China), according to the manufacturer’s instructions. The rate of apoptosis was analyzed using the dual laser flow cytometer (Becton Dickinson, San Jose, CA, U.S.A.) and estimated using the ModFit LT software (Verity Software House, Topsham, ME, U.S.A.).

### Cell migration assay (scratch assay)

HepG2 and SMMC7721 cells were plated on 6-well plates and were allowed to grow until 90% of the cells were confluent. Using a 1 ml pipette tip, a line was scratched on the top of the cells layer. The plates were examined immediately and 24 h after the scratching was performed. The cell migration rate was calculated based on the movement of cells from the initial point to the final distance travelled by the migratory cells during the 24-h incubation period.

### Cell invasion assay

A transwell (8-mm pore size; Millipore, Bedford, MA, U.S.A.) was used for evaluating the cell invasion ability of HCC cells. The upper chamber of the transwell was initially coated with Matrigel (BD Biosciences, Franklin Lakes, NJ, U.S.A.). Serum-free medium and complete medium were added to the upper and lower chambers, respectively. After culturing the cells for 24 h, the top of the membrane was wiped off and the bottom layer of the cells were stained using 0.1% Crystal Violet prepared in 4% paraformaldehyde (PFA). The invading cells were then quantified by counting the cells in 10 random fields under a light microscope (E200; Nikon Corporation, Tokyo, Japan).

### Colony formation assay

HCC cells were cultured in complete culture medium for about 10 days until the colonies were clearly observed. Colonies were fixed with methanol for 10 min and then stained with 0.1% Crystal Violet solution (Sigma-Aldrich) for 5 min. The colonies were counted using the ImageJ software.

### RNA pull-down assay

To determine the interaction between RP11-284P20.2 and *c-met* mRNA, the RNA corresponding to RP11-284P20.2 (sense) and RP11-284P20.2-AS (anti-sense) regions were *in vitro* transcribed and biotin-labeled using T7 RNA polymerase (Roche) and Biotin RNA Labeling Mix (Roche). RP11-284P20.2 RNAs were incubated with the HCC cell lysates and extracted using streptavidin-coupled magnetic beads provided in the Pierce™ Magnetic RNA Pull-Down Kit (Pierce, Rockford, IL, U.S.A.) according to the manufacturer’s instructions. RNA–RNA complexes were eluted using salt solution and purified using TRIzol reagent. qPCR was performed to detect the enrichment of *c-met* mRNA in the retrieved RNA–RNA complexes.

After being treated with DNase I (TaKaRa, Japan) to remove DNA and purified using RNeasy Mini Kit (Qiagen, Shenzhen, China), 1 mg of total RNA was incubated with 3 mg of RP11-284P20.2 (sense) and RP11-284P20.2-AS (anti-sense) for overnight at 4°C. To determine the interaction between RP11-284P20.2 and the eukaryotic translation initiation factor 3b (EIF3b), RNA–protein complexes were isolated using the streptavidin agarose beads (Invitrogen, U.S.A.). The EIF3b protein in the RNA–protein complex was detected using Western blotting as described above.

### RNA immunoprecipitation (RIP)

HepG2 and SMMC7721 cells were lysed using the RIP buffer (Millipore, Billerica, MA, U.S.A.). After centrifugation, the supernatants were incubated with magnetic beads conjugated with anti-EIF3b antibody (Santa Cruz Biotechnology, CA, U.S.A.) for overnight at 4°C. Non-conjugated mouse IgG (Millipore, Billerica, MA, U.S.A.) was used as a negative control. Finally, the isolated and purified RNAs were further used to quantify RP11-284P20.2 using the qPCR analysis.

### RNA fluorescence *in situ* hybridization (FISH) and immunofluorescence (IF)

In the present study, we performed RNA FISH assay in combination with immunofluorescence (IF) detection to identify if the lncRNA RP11-284P20.2 and EIF3b protein colocalizes with each other. The RNA oligonucleotides (probes:5′-AGTGGGTGATTGACTCGGTGAACGCT-3′) targeting RP11-284P20.2 were pre-designed (Biosearch Technologies Inc., Petaluma, CA, U.S.A.). HepG2 and SMMC7721 cells were initially fixed using 3.7% formaldehyde in PBS (pH 7.4) for 10 min at room temperature and then permeabilized using 70% ethyl alcohol at 4°C for least 1 h. Probes were allowed to hybridize to the fixed cells according to the manufacturer’s instructions (Biosearch Technologies Inc., Petaluma, CA, U.S.A.). Later, an anti-EIF3b primary antibody (1:1000 dilution; Santa Cruz Biotechnology) was applied to the cells followed by an incubation step for at least 2 h at 37°C. In the end, the cells were stained with DAPI (4′,6-diamidino-2-phenylindole; Sigma, St Louis, MO, U.S.A.) and the examined using a laser confocal microscope (Leica SP5, Heidelberg, Germany).

### Statistical analyses

The results are presented as the mean ± SD of three independent biological replicates. An intergroup comparison was carried out using the Student’s *t*-test and the statistical variance was estimated using the one-way analysis of variance (ANOVA) test (SPSS13.0 software; Chicago, IL, U.S.A.), according to data type. Correlation analysis was performed using the Spearman’s test. Chi square test or Fisher exact probability methods were used to determine the relationship between the level of RP11-284P20.2 and the clinicopathological characteristics of HCC. When *P* < 0.05, the differences were considered to be statistically significant.

## Results

### Up-regulated expression of RP11-284P20.2 is correlated with an increased expression of c-met protein in HCC tissue

**Figure 1:**
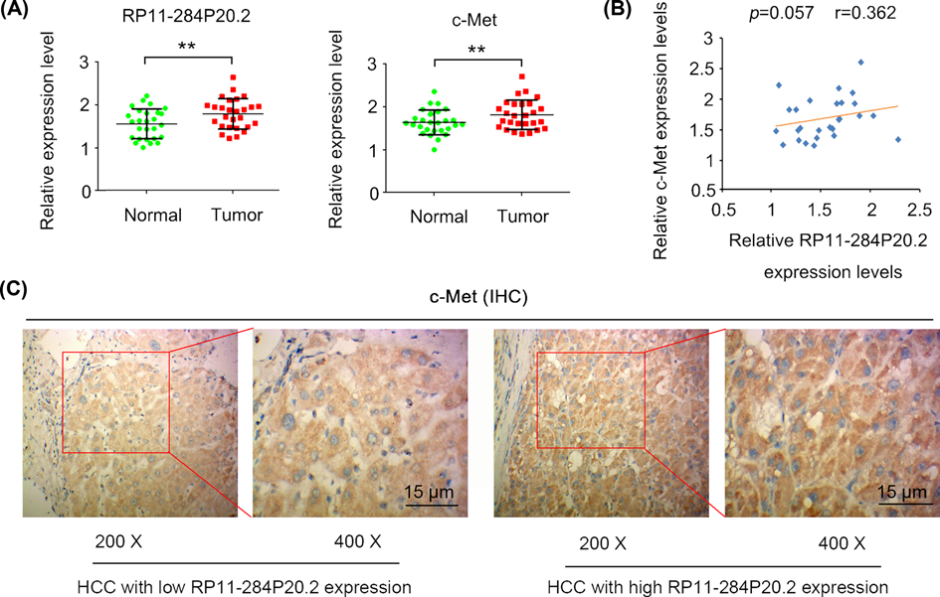
Up-regulated expression of RP11-284P20.2 is correlated with an increased expression of c-met protein in HCC tissue (**A**) The present study quantified the expression level of RP11-284P20.2 and *c-met* mRNA in the HCC and the adjacent paracancerous tissues using qPCR analysis. (**B**) No significant correlation was observed between the RP11-284P20.2 and *c-met* mRNA expression levels as indicated by Spearman analysis. (**C**) According to the average expression level of RP11-284P20.2, HCC tissues were segregated into two groups, one with high (*n* = 13) and another with low RP11-284P20.2 expression level (*n* = 14), respectively. The present study performed histopathological examination of the HCC tissues with low and high RP11-284P20.2 expression levels. Here, we found that the HCC tissues with high RP11-284P20.2 expression level exhibited an increase in c-met protein level compared with the HCC tissues with low RP11-284P20.2 expression. **P < 0.01 versus normal tissues.

In the present study, we initially quantified the expression level of RP11-284P20.2 and *c-met* mRNA in the HCC and the adjacent paracancerous tissues using qPCR analysis. We observed a significant up-regulation in the expression of both RP11-284P20.2 and *c-met* mRNA in HCC tissues (*P* < 0.01, Figure 1A). According to the average expression level of RP11-284P20.2, HCC tissues were segregated into two groups, one with high (*n* = 13) and another with low RP11-284P20.2 expression level (*n* = 14), respectively. HCC with high expression of RP11-284P20.2 was associated with bigger tumor size of HCC (> 5 cm, *P* < 0.05) than HCC with low expression of RP11-284P20.2 (Table 1). No significant correlation was observed between the RP11-284P20.2 and *c-met* mRNA expression levels as indicated by Spearman analysis (*P =* 0.057, *r* = 0.362; Figure 1B). We also performed histopathological examination of the HCC tissues with low and high RP11-284P20.2 expression levels. Here, we found that the HCC tissues with high RP11-284P20.2 expression level exhibited an increase in c-met protein level compared with the HCC tissues with low RP11-284P20.2 expression (Figure 1C).

**Table 1:** Relationship between RP11-284P20.2 expression and the clinicopathological features of patients with HCC

Clinicopathological features	*n*	High expression	Low expression	*P* value
Gender				0.918
Male	21	10	11	
Female	6	3	3	
Age (years)				0.744
≤50	7	3	4	
>50	20	10	10	
Tumor size (cm)				0.011
≤5	17	5	12	
>5	10	8	2	
Metastasis				0.085
Yes	12	8	4	
No	15	5	10	
Clinical stage				0.081
I/II	13	4	9	
III	14	9	5	

When *P* < 0.05, the differences were considered to be statistically significant.

### Depletion of RP11-284P20.2 leads to a decrease in c-met protein expression level in HCC cells

**Figure 2:**
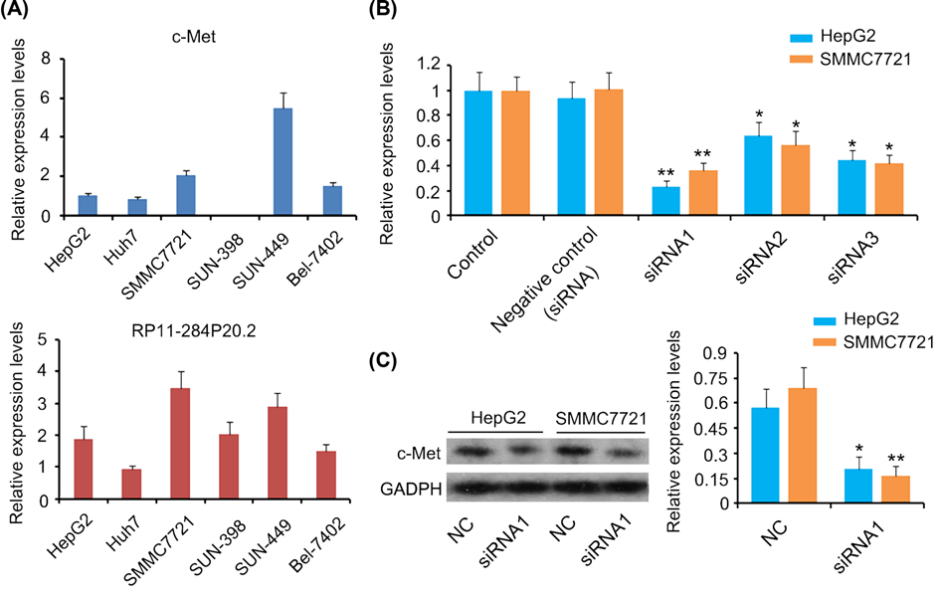
Depletion of RP11-284P20.2 decreased c-met protein expression level in HCC cells (**A**) qPCR analysis was performed to evaluate the expression of RP11-284P20.2 and *c-met* mRNA in different HCC cell lines including HepG2, Huh7, SMMC7721, SUN-398, SUN-449, and Bel-7402. (**B**) PCR was performed to evaluate the expression of RP11-284P20.2 in HCC cells upon transfection with three different RP11-284P20.2-siRNAs. (**C**) Western blot was performed to evaluate *c-met* protein level in HCC cells after RP11-284P20.2 knockdown. *P < 0.05 and **P < 0.01 versus control (**B**) or negative control (**C**).

We performed qPCR analysis to evaluate the expression of RP11-284P20.2 and *c-met* mRNA in different HCC cell lines including HepG2, Huh7, SMMC7721, SUN-398, SUN-449, and Bel-7402. We observed that the HCC cell line SUN-449 exhibited the highest expression of *c-met* mRNA, while SUN-398 exhibited the lowest *c-met* mRNA expression level among all the tested cell lines (Figure 2A). These results indicate that the *c-met* mRNA expression is highly variable in SUN cell lines. Moreover, HepG2 and SMMC7721 cell lines exhibited medium expression levels of *c-met* mRNA among these HCC cell lines. We also observed that the SMMC7721 cells exhibited the highest expression of RP11-284P20.2, followed by SUN-449 and HepG2 cells. In the present study, we further investigated the effect of RP11-284P20.2 on c-met protein expression levels in HepG2 and SMMC7721 cells. Thus, upon transfection with three different RP11-284P20.2-siRNAs, we observed down-regulation of RP11-284P20.2 expression, with the most profound reduction in the expression of RP11-284P20.2 caused by RP11-284P20.2-siRNA1 (*P* < 0.01, Figure 2B). The knockdown of RP11-284P20.2 by siRNA1 was significantly correlated with c-met protein expression levels in HepG2 (*P* < 0.05) and SMMC7721 cells (*P* < 0.01, Figure 2C), but had no significant correlation with *c-met* mRNA expression level (data not shown).

### RP11-284P20.2 has regulatory effects on various characteristics of HCC cells

**Figure 3:**
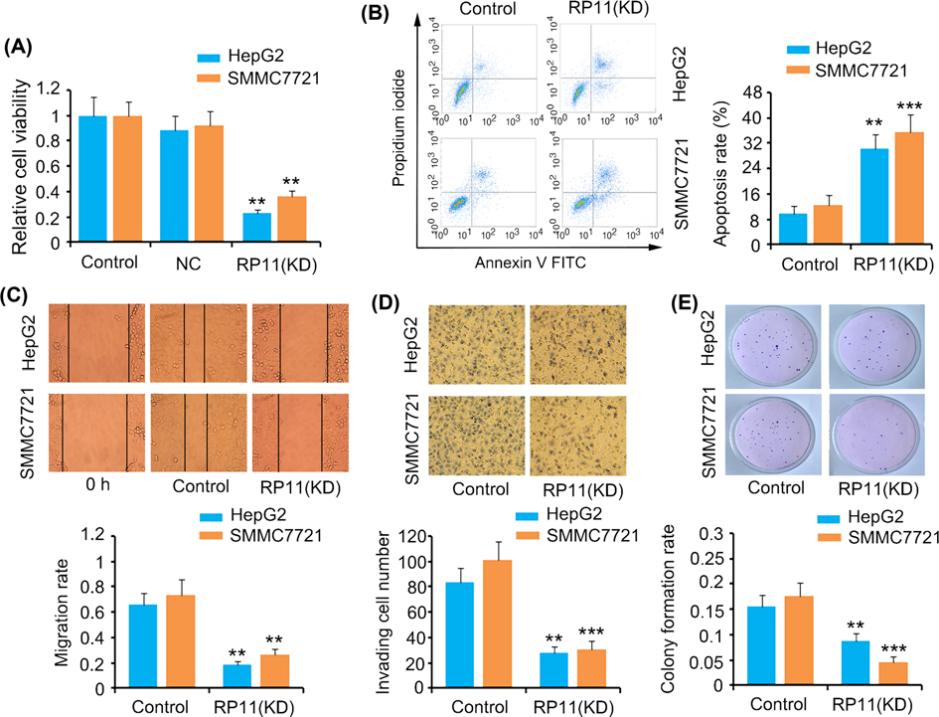
RP11-284P20.2 modulated various characteristics of HCC cells RP11-284P20.2 was knocked down in HepG2 and SMMC7721 cells, followed by the evaluation of cell viability (**A**), apoptosis (**B**), cell migration (**C**), invasion (**D**), and colony formation (**E**). **P < 0.01 and ***P < 0.001 versus control.

Since RP11-284P20.2 is correlated with the c-met protein expression level in HCC cells, it is possible that RP11-284P20.2 modulates various characteristics of HCC cells, including cell viability, apoptosis, cell migration, invasion, and colony formation. Thus, upon RP11-284P20.2 knockdown, a dramatic decrease was observed in the cell viability of HepG2 and SMMC7721 (*P* < 0.01, Figure 3A). Conversely, rate of apoptosis of HepG2 and SMMC7721 cells increased upon depletion of RP11-284P20.2 (*P* < 0.01 or *P* < 0.001, Figure 3B). However, silencing of RP11-284P20.2 inhibited the migration and invasion of HepG2 and SMMC7721 cells (*P* < 0.01 or *P* < 0.001, Figure 3C,D). The colony formation rate of HepG2 and SMMC7721 cells was also undermined upon RP11-284P20.2 knockdown (*P* < 0.01 or *P* < 0.001, Figure 3E).

### RP11-284P20.2 induces c-met protein enrichment by recruiting EIF3b

**Figure 4:**
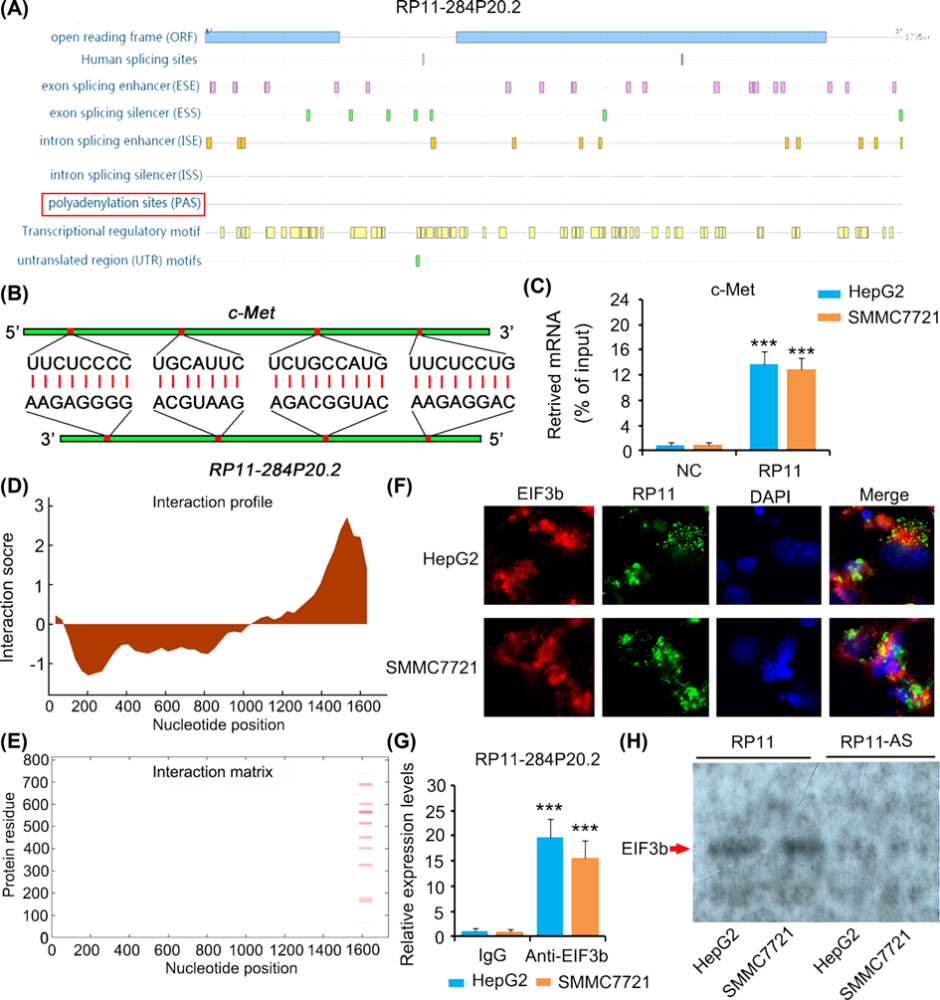
RP11-284P20.2 induces c-met protein enrichment by recruiting EIF3b (**A**) Bioinformatics analysis (http://annolnc.cbi.pku.edu.cn/) showed that RP11-284P20.2 contains several open reading frames; however, it lacks polyadenylation sites. (**B**) Using bioinformatics analysis (http://rna.informatik.uni-freiburg.de/IntaRNA/Input.jsp), we identified an interaction between RP11-284P20.2 and *c-met* mRNA via complementary base pairing. (**C**) RNA pull-down assay was performed to confirm the interaction between RP11-284P20.2 and *c-met* mRNA. ***P < 0.001 vs. NC (negative control) (**D**) Bioinformatics analysis (http://service.tartaglialab.com/page/catrapid_group) revealed an interaction between RP11-284P20.2 and EIF3b protein. (**E**) As indicated by the interaction matrix, the sequence near the 1600 bp locus of RP11-284P20.2 had high affinity toward the region of 200–700 amino acids of EIF3b protein. (**F**) In the results obtained from the FISH + IF assay, we observed an overlapping fluorescence signal from both, RP11-284P20.2 and EIF3b. The present study detected RP11-284P20.2 in EIF3b protein–RNA complex in the RIP assay (**G**) and EIF3b protein in RP11-284P20.2-protein complex in the RNA pull-down assay (**H**). ***P < 0.001 versus IgG group.

Bioinformatics analysis (http://annolnc.cbi.pku.edu.cn/) showed that RP11-284P20.2 contains several open reading frames; however, it lacks polyadenylation sites, which is probably an importance cause that RP11-284P20.2 is unable to be translated to protein (Figure 4A). Using bioinformatics analysis (http://rna.informatik.uni-freiburg.de/IntaRNA/Input.jsp), we identified an interaction between RP11-284P20.2 and *c-met* mRNA via complementary base pairing (Figure 4B). We further performed RNA pull-down assay to confirm the interaction between RP11-284P20.2 and *c-met* mRNA (Figure 4C). Bioinformatics analysis (http://service.tartaglialab.com/page/catrapid_group) also revealed an interaction between RP11-284P20.2 and EIF3b protein (Figure 4D). As indicated by the interaction matrix, the sequence near the 1600 bp locus of RP11-284P20.2 had high affinity towards the region of 200–700 amino acids of EIF3b protein (Figure 4E). To determine and confirm the interaction between RP11-284P20.2 and EIF3b protein, we performed FISH + IF, RIP and RNA pull-down assays. In the results obtained from the FISH + IF assay, we observed an overlapping fluorescence signal from both, RP11-284P20.2 and EIF3b (Figure 4F). Moreover, we also detected RP11-284P20.2 in EIF3b protein–RNA complex in the RIP assay (Figure 4G) and EIF3b protein in RP11-284P20.2–protein complex in the RNA pull-down assay (Figure 4H).

### The regulatory effect of RP11-284P20.2 on HCC growth properties is dependent on c-met protein expression level

**Figure 5:**
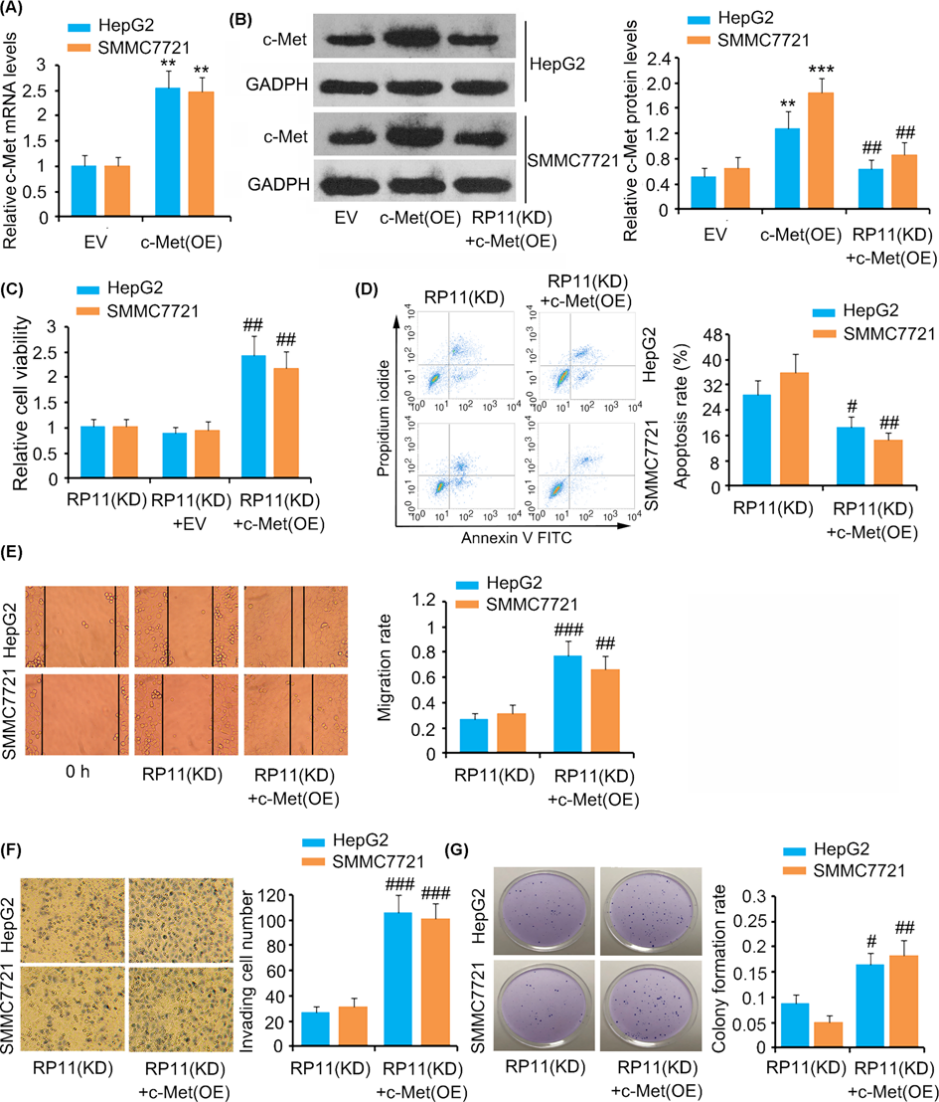
The regulatory effect of RP11-284P20.2 on HCC growth properties is dependent on c-met protein expression level (**A**) qPCR analysis was performed to evaluate the expression of *c-met* mRNA in HCC cells after transfecting the expression vectors. (**B**) Western blot was performed to evaluate *c-met* protein level in HCC cells after transfecting *c-met* expression vectors alone or in combination with RP11-284P20.2-siRNA. The present study evaluated cell viability (**C**), apoptosis (**D**), cell migration (**E**), invasion (**F**), and colony formation (**G**), after transfecting *c-met* expression vectors alone or in combination with RP11-284P20.2-siRNA. EV: expression vector. KD: knockdown. **P < 0.01 and ***P < 0.001 versus EV group (*c-met* overexpression). ^#^*P* < 0.05, ^##^*P* < 0.01 and ^###^*P* < 0.001 vs. RP11-284P20.2 knockdown group.

We performed rescue experiments to determine whether the regulatory effects of RP11-284P20.2 on HCC growth properties are dependent on c-met. We observed that transfection of HepG2 and SMMC7721 cells with the c-met expression vector lead to an increase in the *c-met* mRNA and c-met protein expression level (*P* < 0.01 or *P* < 0.001, Figure 5A,B). However, silencing of RP11-284P20.2 attenuated the increase of c-met protein expression level caused the expression vesctor (*P* < 0.01 versus c-met overexpression group, Figure 5B). Further, we also observed that RP11-284P20.2 knockdown combined with c-met overexpression promoted cell viability (*P* < 0.01, Figure 5C), suppressed apoptosis rate (*P* < 0.05 or *P* < 0.01, Figure 5D), and elevates cell migration (*P* < 0.01 or *P* < 0.001, Figure 5E), invasion (*P* < 0.001, Figure 5F), and colony formation rates (*P* < 0.05 or *P* < 0.01, Figure 5G), compared with RP11-284P20.2 knockdown alone.

## Discussion

We found that the lncRNA RP11-284P20.2 promotes the growth and invasion of HCC cells. Furthermore, we revealed that the cancer-promoting effects of RP11-284P20.2 are associated with the up-regulation of c-met protein expression level. *RP11-284P20.2* gene contains several open reading frames, but it lacks polyadenylation site, a key sequence recognized by polyadenylate polymerase, which is responsible for adding polyadenylation [poly(A)] tail to the 3′UTR of mRNA. Poly(A) tail prevents the degradation of mRNA and promotes its transport from nucleus to cytoplasm for further translation process [7,8]. Without poly(A) tail, it is assumed that the lncRNA RP11-284P20.2 can be readily degraded in most of the cells. However, we found that the expression level of RP11-284P20.2 was notably up-regulated in HCC tissues, while the underlying mechanism that resulted in this up-regulation is unclear. LncRNA MALAT1 and MEN β also lack poly(A) tails; however, they form a special triple helical structure that stabilizes such two lncRBAs [9]. Since RP11-284P20.2 is a pseudogene, which cannot be translated to a protein, thus it is classified as an lncRNA.

Recent reports have unveiled several lncRNAs that play oncogenic or tumor-suppressive roles in HCC. For instance, a lncRNA named LOC105369748 was found to overexpress in HCC tissues and further promote the proliferation, migration, invasion, and epithelial-to-mesenchymal transition of HCC cells [10]. Similarly, FLVCR1 antisense RNA 1 (FLVCR1-AS1) is another lncRNA was reported to be up-regulated in HCC tissues. FLVCR1-AS1 was shown to act as a sponge for the miRNA-513c, which in turn leads to an increase in c-met expression level, resulting in enhanced cell proliferation and invasion of HCC cells [11]. In contrast, lncRNA DGCR5 (DiGeorge syndrome critical region gene 5) was found to be down-regulated in HCC tissues. Overexpressing the lncRNA DGCR5 inhibits the development of HCC by targeting the miRNA–346/KLF14 axis [12]. In the present study, we demonstrated that the knockdown of RP11-284P20.2 is associated with suppressed viability, migration, invasion and colony formation and increased apoptosis of HCC cells. These data suggest that RP11-284P20.2 promotes HCC development.

During the histopathological examination, we found that the HCC tissues with high RP11-284P20.2 expression exhibits significantly higher c-met protein expression level compared with the HCC tissues with low RP11-284P20.2 expression. However, we did not observe positive correlation between RP11-284P20.2 and *c-met* mRNA expression level. These data suggest that RP11-284P20.2 is associated with the regulation of c-met protein expression level rather than *c-met* mRNA expression level. Various biological functions of lncRNAs have been identified. For instance, lncRNAs can act as competing endogenous RNAs (ceRNA) of endogenous miRNAs, which prevents the binding of the miRNAs to their target mRNAs [12]. In addition, lncRNAs are also associated with epigenetic regulation as they can interact with several methylases and acetylases [13]. These regulatory functions of lncRNAs influence the mRNA expression of the target genes, consequently modulating their protein expression levels. Moreover, lncRNAs are able to affect protein expression level independently, without influencing the mRNA expression level, by predominantly regulating mRNA translation and protein stability [14,15]. In the present study, we identified that the lncRNA RP11-284P20.2 interacts with *c-met* mRNA via complementary base pairing, and also found that RP11-284P20.2 binds to EIF3b protein with high affinity. Since EIF3b is required for several steps in the initiation of protein synthesis, we could state that with the help of RP11-284P20.2, EIF3b interacts with *c-met* mRNA, thereby promoting c-met protein synthesis.

Furthermore, a few of studies have reported that lncRNAs are involved in the regulation of mRNA translation. Zhang et al. reported that the lncRNA ARAP1-AS1 drives the translation of proto-oncogene c-myc by directly interacting with PSF protein and further affect the internal ribosome entry site (IRES) function. Up-regulation of c-myc driven by ARAP1-AS1 facilitates the development and progression of cervical cancer [16]. Another lncRNA GMAN is shown to directly interact with EIF4b and promote its phosphorylation and stability [17]. As a result, the translation of several anti-apoptosis-related proteins was enhanced. In our study, we found that the lncRNA RP11-284P20.2 recruits EIF3b to *c-met* mRNA, which probably induces *c-met* translation. However, we did not knockdown EIF3b to further determine whether the regulatory effect of RP11-284P20.2 on c-met is dependent on EIF3b. Since EIF3b is a key protein required during the translation process, silencing of EIF3b could lead to the dysfunctional translation of a large number of mRNAs.

Further, to determine whether the effects of RP11-284P20.2 on multiple properties of HCC rely on c-met protein expression, we performed a rescue experiment by overexpressing c-met along with RP11-284P20.2 knockdown. We observed that an increase in c-met expression restored the viability, migration, invasion, and colony formation abilities of the HCC cells, which were undermined upon RP11-284P20.2 knockdown. These data suggest that c-met mediates most of the cancer-promoting effects of HCC.

In summary, we found that the lncRNA RP11-284P20.2 promotes growth and invasion of HCC cells. Using FISH+IF, RIP, and RNA pull-down assays, we revealed that RP11-284P20.2 facilitates the interaction between EIF3b and *c-met* mRNA, which probably leads to an enhanced c-met protein synthesis. With the help of a rescue experiment, we also confirmed that c-met mediates most of the cancer-promoting effects of HCC. Therefore, we conclude that the lncRNA RP11-284P20.2 promotes cell proliferation and invasion in HCC by recruiting EIF3b to enhance c-met translation.

## Data Availability

The datasets generated/analyzed in the present study are available upon reasonable request from the corresponding author.

## Abbreviations

EIF3b: eukaryotic translation initiation factor 3b
FISH: fluorescence *in situ* hybridization
HCC: hepatocellular carcinoma
HGF: hepatocyte growth factor
IF: immunofluorescence
lncRNA: long non-coding RNA
ncRNA: non-coding RNA
RIP: RNA immunoprecipitation
